# Plant Health and Rhizosphere Microbiome: Effects of the Bionematicide *Aphanocladium album* in Tomato Plants Infested by *Meloidogyne javanica*

**DOI:** 10.3390/microorganisms8121922

**Published:** 2020-12-03

**Authors:** Claudia Leoni, Elisabetta Piancone, Nicola Sasanelli, Giovanni Luigi Bruno, Caterina Manzari, Graziano Pesole, Luigi R. Ceci, Mariateresa Volpicella

**Affiliations:** 1 Institute of Biomembranes, Bioenergetics and Molecular Biotechnologies, CNR, Via Amendola 165/A, 70126 Bari, Italy; c.leoni@ibiom.cnr.it (C.L.); c.manzari@ibiom.cnr.it (C.M.); graziano.pesole@uniba.it (G.P.); 2 Department of Biosciences, Biotechnologies and Biopharmaceutics, University of Bari Aldo Moro, Via Amendola 165/A, 70126 Bari, Italy; e.piancone@ibiom.cnr.it; 3 Institute for Sustainable Plant Protection, CNR, Via G. Amendola 122/D, 70126 Bari, Italy; nicola.sasanelli@ipsp.cnr.it; 4 Department of Soil, Plant and Food Sciences, University of Bari Aldo Moro, Via Amendola 165/A, 70126 Bari, Italy; giovanniluigi.bruno@uniba.it; 5 Consorzio Interuniversitario Biotecnologie, Località Padriciano, 99, Area di Ricerca, 34149 Trieste, Italy

**Keywords:** rhizosphere, microbiome, *Aphanocladium album*, *Meloidogyne javanica*, biocontrol agent

## Abstract

The artificial introduction in the soil of antagonistic microorganisms can be a successful strategy, alternative to agrochemicals, for the control of the root-knot nematodes (*Meloidogyne* spp.) and for preserving plant health. On the other hand, plant roots and the associated rhizosphere constitute a complex system in which the contribution of microbial community is fundamental to plant health and development, since microbes may convert organic and inorganic substances into available plant nutrients. In the present study, the potential nematicidal activity of the biopesticide *Aphanocladium album* (*A. album* strain MX-95) against the root-knot nematode *Meloidogyne javanica* in infected tomato plants was investigated. Specifically, the effect of the *A. album* treatment on plant fitness was evaluated observing the plant morphological traits and also considering the nematode propagation parameters, the *A. album* MX-95 vitality and population density. In addition, the treatment effects on the rhizosphere microbiome were analysed by a metabarcoding procedure. Treatments with *A. album* isolate MX-95 significantly decreased root gall severity index and soil nematode population. The treatment also resulted in increased rhizosphere microbial populations. *A. album MX-95* can be favourably considered as a new bionematicide to control *M. javanica* infestation.

## 1. Introduction

Root-knot nematodes are widespread and polyphagous pests, which cause severe yield losses to numerous vegetables [1]. In general, the control of nematode pests is based on the use of chemical methods, such as fumigants, which ensure a rapid and effective response [2]. However, deleterious effects of pesticides on human and animal health as well as the high cost and the environmental risks related to their use, strongly evoke alternative and eco-friendly strategies for parasites management. In this perspective, the artificial introduction of microorganisms for controlling antagonists of plant development can be extremely useful in those cases in which the chemical control is not economic and unreasonably harmful for human healthy or when pesticides fail against pests because of their resistance development. In the case of nematode parasites, fungi have been extensively studied and applied [3]. For example, fungi such as *Aspergillus niger* Tiegh., *Purpureocillium lilacinum* (Thom) Luangsa-ard, Houbraken, Hywel-Jones and Samson (syn *Paecilomyces lilacinus* (Thom) Samson), *Trichoderma harzianum* Rifai, *T. viride* Pers. and *Metacordyceps chlamydosporia* (H.C. Evans) G.H. Sung, J.M. Sung, Hywel-Jones and Spatafora are routinely used as bionematicides applied against plant-parasitic nematodes [4]. They are worldwide diffuse microorganisms belonging to diverse phylogenetic groups (Ascomycota, Basidiomycota, Zygomycota and Chytridiomycota). Some carnivorous fungi, under adverse nutritional conditions, can also switch their feeding on nematodes [5]. The strain MX-95 of the fungus *Aphanocladium album* (Preuss) W. Gams has shown appreciable effects in the biological control of powdery mildew of tomato, squash and cucumber, caused by *Oidium lycopersici* L. Kiss and *Spaerotheca fusca* Blumer [6,7]. The fungus produces hydrolytic enzymes [8], such as chitinases, which are responsible for the total or partial degradation of cell walls of numerous plant pathogens and parasites.

However, plant rhizosphere is a complex environment in which around 10^11^ microbial cells per gram root are present, with more than 33,000 bacterial and archaeal species [9,10]. Their characterization by standard culturing laboratory techniques is almost impossible, making it difficult to understand their interactions with complex environments. On the other hand, recent advances in NGS technologies and computational tools allow to carry out an increasing number of metagenomic studies in different environments, useful to provide key insights regarding the relationships between the host and the microbial communities [11,12]. Plants can modify the bacterial population of their rhizosphere shaping a beneficial microbe community able to increase the resistance against soil borne pathogens [9]. Kwak et al., using a metabarcoding and a shotgun sequencing of the whole genome from the rhizosphere or bulk soil, found that disease-resistant tomatoes recruit bacterial allies to protect themselves from infection [13].

Although details of the possible (and contrasting) effects of the rhizosphere microbiota during nematode infections have already been described, little is known about the dynamics of the rhizosphere microbiota during nematicide treatments [14,15,16,17]. A broader understanding of the plant-microbe interactions may help in establishing better plant nematicide treatments [18,19]. In this study, the potential nematicide activity of the promising biopesticide *A. album* (strain MX-95) against the root-knot nematode *Meloidogyne javanica* (Treub) Chitw. was evaluated in infested tomato plants. At the same time, the rhizosphere microbiomes were analysed and described.

## 2. Materials and Methods

### 2.1. Glasshouse Experiment

Clay pots (V = 1000 mL) filled with a sandy soil (sand >99%; silt <1%; clay <1% and organic matter = 0.75%; pH 7.2) were arranged on benches, in a glasshouse at 25 ± 2 °C, according to a randomized block design. In each pot a single seedling of one-month-old tomato (*Solanum lycopersicum* L.) cv. Roma VF was transplanted, on which an Italian population of *M. javanica* was reared for sixty days. Tomato plants were then uprooted, and from infected roots, previously gently washed, egg-masses were handpicked with the help of a stereomicroscope. Batches of twenty egg-masses of similar size (averaging 20,000 eggs) were placed on 2 cm diameter sieves (215 µm aperture), and each sieve was put in a 3.5 cm diameter Petri dish. Three millilitres of distilled water (natural hatching agent), sufficient to cover egg-masses was then added to the batches of egg-masses. The dishes were incubated in a growth cabinet at 25 ± 2 °C to obtain *M. javanica* juveniles (J2)—that is, the infective stage of the nematode to use in the experiment [20].

Treatments were: (i) tomato cropped in uninfested soil (Control); (ii) tomato + *M. javanica* infested soil (N); (iii) N treated with *A. album* strain MX-95 (N + MX95); (iv) tomato + uninfested soil treated with *A. album* strain MX-95 (MX95) and (v) N treated with Tervigo (N + Ter). For each treatment, 5 biological replicates were performed. The strain MX-95 of *A. album* was grown on Potato-Dextrose-Agar (PDA) in Petri dishes in the dark at 24 °C ± 1 °C for 12 days. Conidia were collected in sterile Potato-Dextrose-Broth amended as surfactants with 2 mL/L of Tween 80 (PDB-T) to allow the dispersion of strongly hygroscopic conidia. About 20 mL of PDB-T was poured, and the plates were swirled handily and gently to favour conidia detachment. The conidia suspension was transferred with a sterile pipette. Then, the concentration of the inoculum was determined and diluted to obtain 1.2 × 10^7^ conidia/mL corresponding to 8.6 × 10^6^ CFU/mL (vitality 95.3%). Because the maximum chitinolytic activity of *A. album* strain MX-95 is developed 3–4 weeks after soil treatments, the fungus was applied three times: 4 weeks before nematode soil infestation; 1 day before tomato transplant; 1 month after transplant. For each MX-95 treatment, the soil was watered with 125 mL and then amended with 25 mL of the conidial suspension of the fungus. Control pots received 25 mL of PDB-T.

Tervigo SC (Syngenta Italia S.p.a., Milan, Italy), a commercial nematicide formulation based on the exametabolite abamectin (20 g/L) produced by *Streptomyces avermitilis* (ex Burg et al.) Kim and Goodfellow [21,22] and the uninfested soil were used as controls.

One-month old tomato seedling cv. Regina was transplanted in each pot (5 April 2019) and 4 days later infested with an initial *M. javanica* population (Pi) of 750 J2.

**Figure 1:**
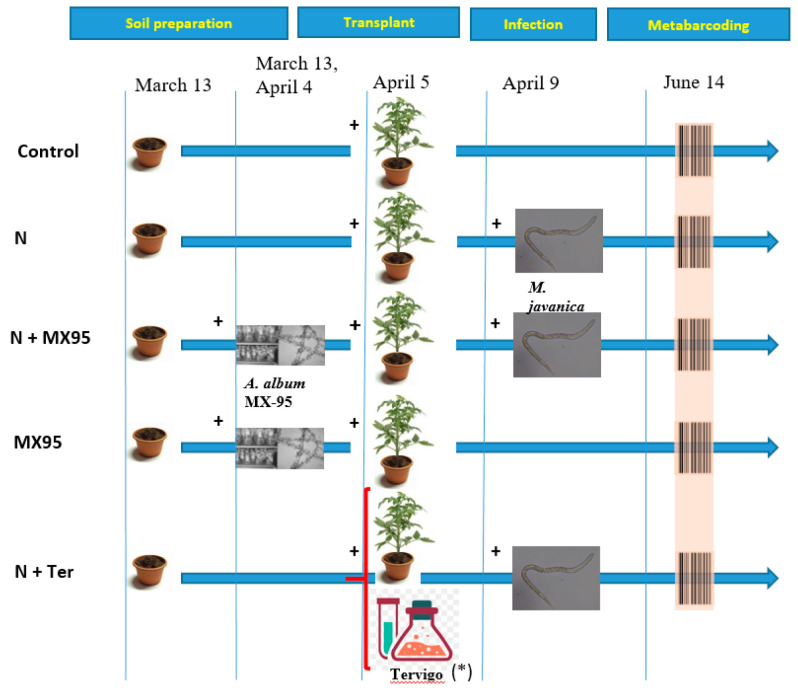
Pot experiment design. The different treatments are shown. Sampling for 16S rRNA gene metabarcoding analysis is indicated by a barcode symbol. (*) Tervigo (also applied on 19 April, 3 and 17 May). Treatments: Control = *S. lycopersicum* cv. Roma plants cropped in uninfested soil; N = tomato plants cropped in soil infested with *M. javanica*; N + MX95 = treatment N with *A. album* strain MX-95 applications; MX95 = tomato plants cropped in uninfested soil treated with MX-95; N + Ter = treatment N plus Tervigo applications.

Tervigo was applied at transplant (5 April) and another 3 times every 14 days (19 April, 3 and 17 May). For Tervigo distributions, the soil of each pot was watered with 50 mL of distilled water and then treated with 100 mL of nematicide aqueous solution (0.06 mL/L) corresponding to the dose (5 L/ha) recommended against root-knot nematodes. A detailed time schedule of the experiment is reported in Figure 1.

During the experiment, all pots were maintained in the glasshouse at 25 ± 2 °C for two months, randomizing the position of the blocks and at the same time repositioning each plant within a block every week, to avoid a block position effect and at the same time the factor position of the plant within the block. Plants received all the necessary maintenance (irrigation, fertilization, etc.).

At the end of the experiment (60 days after transplantation), tomato plants were uprooted. Height, dry top and root weight were recorded. The severity of nematode root infestation (root gall index (RGI)) was estimated according to a 0–5 scale (0 no galls and 5 root system completely deformed by large and numerous galls) [23].

Soil nematode population density in each pot was determined by processing 500 mL soil by the Coolen’s method [24]. Numbers of *M. javanica* eggs and J2 in roots were assessed by cutting up each root system into small pieces and further grinding with a blender, containing aqueous 1% NaOCl for 20 s [23]. The water suspension was then sieved through a 250 µm pore sieve put over a 22 µm pore sieve. Nematodes and root debris gathered on the 22 µm pore sieve were further processed by centrifuging at 2000 rpm for five min in 400 mL of a magnesium sulphate solution at 1.16 specific gravity. Then eggs and J2 in the water suspension were sieved through the 22 µm pore sieve, sprayed with tap water to wash away the magnesium sulphate solution and collected in about 30–40 mL water. Then, they were counted and final nematode population density (*Pf*) in each pot was determined by summing nematodes recovered from soil and roots. The nematode reproduction factor *r* was expressed as the ratio between final and initial population density (*Pf*/*Pi*) of *M. javanica*.

The isolation of *A. album* and other fungi from soil around root systems was carried out at the end of the experiment. The serial dilution plating method on Rose-Bengal-Malt-Extract-Agar was used [25]. The dilution of soil sample was conducted in two replicates. About 50 g of air-dried soil was added in 100 mL of sterile suspending solution (SS: 0.8% NaCl and 0.2% technical agar) and stirred for 20 min to mix the solution. The solution was diluted to ten series of decimal dilution with SS. For each dilution, 1 mL was spread on each Petri dish. Plates (two for each dilution) were incubated at 25 °C for ten days. The colonies of fungi that appeared in the plate after incubation were transferred on a PDA plate incubated at 25 °C for 7 days and identified on the basis of their morphological features.

### 2.2. DNA Extraction, Amplicon Library Preparation and Illumina-Based Sequencing

At 60 days after transplantation, plants were uprooted. In order to remove bulk soil, plants were vigorously shaken by hand, paying attention to the integrity of the roots, until non-adhering soil particles were completely removed from roots. Rhizosphere soil samples were afterwards collected in sterile falcons, immediately frozen in liquid nitrogen and stored at −80 °C [26]. Additionally, sandy soil samples were also collected before the transfer into the pots, in order to verify the sterility of the soil used.

Total DNA was extracted from each sample, starting from 0.5 gr of soil using the FastDNA SPIN Kit for Soil (MP Biomedicals, Irvine, CA, USA), according to the manufacturer’s instructions and as already reported [27]. DNA was eluted in 100 µL of sterile water and stored at −20 °C until further analysis. The quality and concentration of the DNA extracts were determined by 1% agarose gel electrophoretic analysis and by spectrophotometric measurements at 260, 280 and 230 nm using a NanoDrop^®^ ND-1000 Spectrophotometer (Thermo Fisher Scientific, Waltham, MA, USA), according to consolidated procedures [27].

Amplicon libraries for bacteria identification were prepared by amplification of the V5-V6 hypervariable region of the 16S rRNA gene, using the primers BV5 (787F): 5′-TCGTCGGCAGCGTCAGATGTGTATAAGAGACAG/ATTAGATACCCYGGTAGTCC-3′ and AV6 (1073R): 5′-GTCTCGTGGGCTCGGAGATGTGTATAAGAGACAG/ACGAGCTGACGACARCCATG-3′ and 0.2 ng of DNA extracted from each rhizosphere sample, according to already established procedures [28,29]. Amplicon libraries were then subjected to a 2 × 250 bp paired-end sequencing using the Illumina MiSeq platform. To increase the genetic diversity, as required by the MiSeq platform, 25% of phage PhiX genomic DNA library was added to the mix and co-sequenced.

### 2.3. Statistical and Bioinformatic Analysis

Data derived from the pot experiment were subjected to analysis of variance (ANOVA) and means compared by a Least Significant Difference Test at *p* ≤ 0.05. All statistical analyses were performed using the PlotIT program Ver. 3.2 (Scientific Programming Enterprises, Haslett, MI, USA). Standard deviation (SD) was also calculated.

The obtained Illumina MiSeq raw data were analysed by using the microbiome bioinformatics platform, QIIME2 2020.2, to generate denoised reads and assign taxonomy [30]. Briefly, raw Paired End (PE) reads were treated with Cutadapt [31] to remove Illumina adaptors, followed by denoising with DADA2 (Divisive Amplicon Denoising Algorithm) workflow [32]. During the denoising step, BV5 (787F) and AV6 (1073R) primers were trimmed, and the raw reads were filtered according to the observed expected error. In order to remove very low abundant amplicon sequence variants (ASVs—features with frequencies lower than 0.00001) and singletons, QIIME2 feature-table filter-features plugin was performed. The obtained ASVs were taxonomically annotated using the QIIME2 plugin fit-classifier-sklearn [33] by using the release 138 of the SILVA database [34] as reference and taxonomy collection. Moreover, QIIME2 taxa filter-table plugin was used to remove all features annotated as mitochondria and chloroplast.

Phylogenetic analysis was performed by using the QIIME2 align-to-tree-mat-fasttree plugin: MAFFT was used to obtain a multiple sequence alignment of ASVs sequences [35] and the maximum-likelihood procedure implemented in Fasttree 2 [36] to infer the phylogenetic tree.

By using MicrobiomeAnalyst [37], rarefaction curve analysis was performed applying the modified function ggrare originated from the ranacapa package2 [38] as implemented in the tool. According to rarefaction curves, the ASV table was normalised for diversity analysis.

The Shannon index (α diversity) was inferred on rarefied ASVs across samples, by applying the R-package phyloseq [39] as implemented in MicrobiomeAnalyst. Statistically relevant differences of grouping based on experimental factors were estimated using the Kruskal–Wallis test; a subsequent Dunn Test for pairwise comparisons was also performed. The principal coordinates analysis (PCoA) describing the diversity between the samples (β-diversity) based on the weighted and unweighted UNIFRAC [40] metrics was inferred by applying the R-package phyloseq [39]. Statistical significance of the clustering pattern was evaluated by using Permutational Multivariate Analysis Of Variance (PERMANOVA) [41] by applying MicrobiomeAnalyst.

High-dimensional metagenomic data were explored using the LDA Effect Size (LEfSe) method, applied at the genus level for all samples. Genera were considered to be significant based on their adjusted *p*-value = 0.05 [42].

Differential abundance analysis at genus level was performed by using the DESeq2 method. Features were considered to be significant based on their adjusted *p*-value (0.05) [43].

## 3. Results and Discussion

### 3.1. Analysis of Plant Morphology and Nematode and Fungus Population Data

Growth parameters of tomato plants recorded in the experiments are reported in Table 1. No significant differences were observed in shoot height of plants grown in *M. javanica* infested soil treated with MX-95 or Tervigo. A significant difference was observed only between the infested and untreated control and the tomato plants cropped in soil treated with MX-95. Dry shoot weight ranged between 16.2 and 19.0 g and was not influenced by the nematode infestation of the soil and by the treatments applied (Table 1). Soil treatment with the *A. album* isolate MX-95 resulted in a significant increase in root weight (166.6 g) higher than those observed in the healthy control (80.2 g) and the infested and untreated control (100.7 g).

Parameters associated to *M. javanica* are shown in Table 2. The RGI recorded for roots of Tervigo treated plants (3.2) was lower than those recorded in the tomato plants cropped in soil infested with *M. javanica* (5.0) and in the MX-95 treated plants (4.0); however, this effect was significant only in the first case. Both nematicides, MX-95 and Tervigo, resulted in a significantly lower number of nematodes extracted from each root system compared to the nematode-treated control but were not statistically different from each other. In plants treated with MX-95, the number of eggs and J2 per gram of root were significantly lower (690) than those observed in plants treated with Tervigo (1570) and in tomato plants cropped in soil infested with *M. javanica* (2150). No statistically significant differences were recorded between the plants treated with Tervigo and the untreated plant. The nematode population density in the soil, calculated as eggs and J2 per soil mL, ranged between 6.1 and 3.3 with any significant difference (Table 2). The treatments with the two nematicides, MX-95 and Tervigo, significantly reduced the total population density of *M. javanica* (*Pf* derived from soil and roots: eggs and J2 per mL soil) in comparison to tomato plants cropped in *M. javanica* infested soil. The highest *M. javanica* reproduction rate (280) was recorded in the N-treated soil. A significant lower reproduction rate was observed in both treatments treated with MX-95 or Tervigo (160 and 238, respectively). Increase in nematode population of infested control plants indicates hosts suitability and tolerance of the cultivar at such levels of *M. javanica* infestation.

The obtained results confirm the potential use of *A. album* isolate MX-95 as an alternative control method, at low environmental impact, against root-knot nematodes, as well as other biocontrol agents commercialized against phytonematodes—i.e., *T. harzianum* [44] and *P. lilacinus* [45,46].

The presence of MX-95 and other fungi in the sampled soil are reported in Table 3. As expected, the strain MX-95 of *A. album* was not isolated from soil used as non-treated control, soil infested with *M. javanica* and soil treated with Tervigo. Furthermore, when inoculated (about 21.1 × 10^3^ CFU per gram of air-dried soil), MX-95 increased its presence in the analysed soil and reached 12 × 10^9^ CFU per gram of air-dried soil. The presence of *M. javanica* in the pots induced a further 3.6-fold increment in MX-95 population. Other fungi, belonging to the *Penicillium* (25%), *Aspergillus* (5%), *Chaetomium* (15%), *Alternaria* (25%), *Mucor* (3%), *Trichoderma* (14%), *Gliocladium* (3%) and other genera (7%), were also isolated.

Under glasshouse-controlled conditions, the bionematicide *A. album* increased colony forming units at the end of the experiments, which is considered an important parameter to evaluate biocontrol potential and performance of a biological control agent [45]. Moreover, the use of *A. album* strain MX95 in soil increased the presence of other fungi, including well known nematode-antagonistic, belonging to the genera *Aspergillus* and *Trichoderma* [47].

### 3.2. Taxonomic Profiles of Rhizosphere by Metabarcoding Analysis

To analyse the bacterial population of the rhizosphere samples, a deep metabarcoding analysis of the 16S rRNA gene was performed using a specific pair of primers targeting the V5–V6 hypervariable region, as already reported [28,29]. Five biological replicates were used for all treatments (Figure 1). The same analysis was not performed on the sandy soil before any treatments because it was not possible to extract any DNA from it, confirming the sterility of the soil before plant transplantation.

For each sample, bacterial amplicons were obtained using 0.2 ng of total DNA. Purified amplicon libraries were sequenced by a 2 × 250 bp paired-end approach on an Illumina MiSeq platform, and about 1,730,211 Illumina MiSeq raw data were obtained.

The raw Paired End (PE) reads were treated to remove Illumina adaptors followed by denoising with DADA2 workflow. On average, 69,208.44 PE (SD 9494.36) were obtained for each sample, and about 79% of them passed the step of denoising (Table S1). All the distinct sequencing raw data are available in the Short Read Archive (SRA) repository (SUB8562371).

The amplicon sequence variants (ASVs) inferred from the denoising step were subsequently filtered to remove very low abundant ASVs (relative abundance lower than 10^−5^). This step allowed the ASVs reduction from 4293 to 2370. Moreover, all features annotated as mitochondria and chloroplast were removed, obtaining finally 2356 ASVs. They were then used to normalize the data by plotting rarefaction curves for each sample (Figure S1). According to the obtained curves, the rarefaction thresholds were set to 40,000, which allowed the adequate sampling of the observed biodiversity and retain almost all the samples, with the exception of one biological replicate from Control treatment (indicate as 1-1A) (Figure S1).

**Figure 2:**
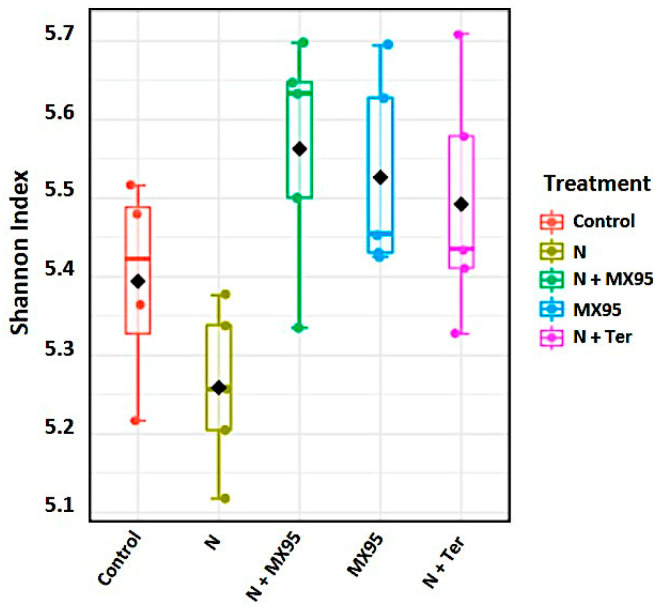
Shannon index values inferred from the 16S rRNA gene amplicon sequences for the assayed treatment. Each treatment is represented with a different colour (see legend). The diversity distribution of a group inside the specific treatment is reported.

The alpha diversity was measured using the Shannon index, and the results are showed as box plots for each treatment (Figure 2). Statistical differences were evaluated using a Kruskal–Wallis test (*p*-value: 0.042481) followed by a Dunn-test (Figure S2).

**Figure 3:**
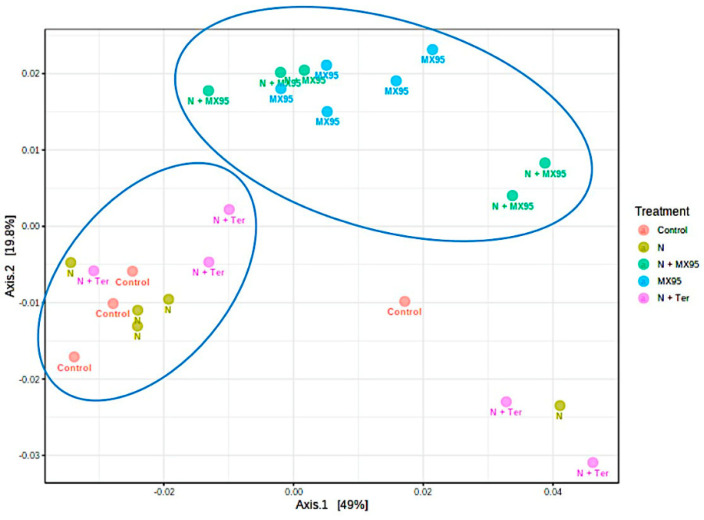
Principal coordinates analysis (PCoA) representation of Weighted Unifrac analysis. Each sample is coloured based on the specific treatment as in the legend; circles surround possible sample clusters.

Beta diversity was measured as Weighted Unifrac and plotted as principal coordinates analysis (PCoA) (Figure 3). A possible cluster is represented by samples in which *A. album* was present (MX95 and N + MX95). Most of the other samples (Control, N and N + Ter) also cluster in a limited area with few samples outside. Statistical significance of the clustering pattern in ordination plots was evaluated using the PERMANOVA test (*F*-value: 2.3193; R-squared: 0.32808; *p*-value < 0.007).

**Figure 4:**
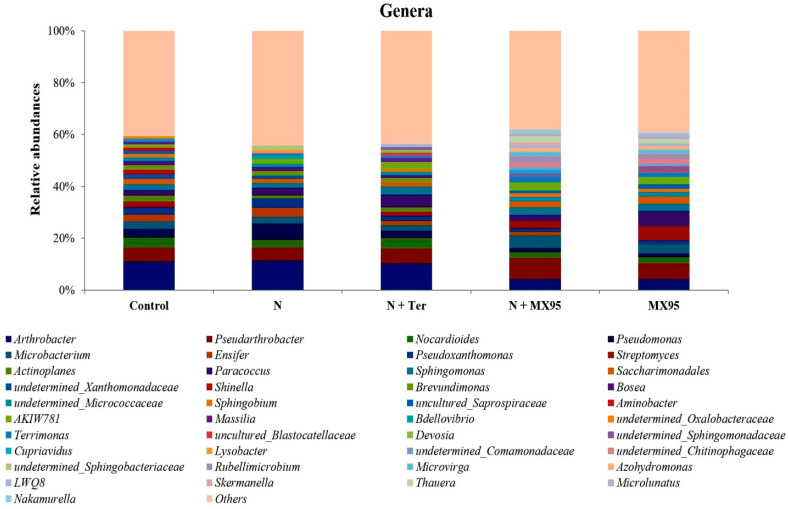
Bar charts of bacteria genera identified in the different treatments.

The taxonomic distribution of bacteria is shown in Figure 4 at genus level. All values were the average of data from the different biological replicates.

Groups with relative abundances < 1.0% were joined as “Others”. ASVs, which could not be resolved at the genus level, were reported with the notation g_uncultured or undetermined followed by the name of the closest known parental rank. A list of the identified genera and their relative abundances is reported in Table S2.

In order to narrow the terms of the discussion, only genera with relative abundances ≥ 1% will be considered from now on. Eleven genera (*Arthrobacter*, *Pseudarthrobacter*, *Nocardioides*, *Pseudomonas*, *Microbacterium*, *Pseudoxanthomonas*, *Paracoccus*, *Sphingomonas*, *Saccharimonadales*, *undetermined_Micrococcaceae* and *AKIW781*) are present in all the samples, with *Arthrobacter* as the only lineage reaching percentages above 10%. Overall, they account for abundances ranging from about 35% (in the two treatments containing *A. album*) to 43% (in the N treatment). Another 4 genera (*Ensifer, Streptomyces*, *Sphingobium* and *Terrimonas*) are present in four of the five treatments. Nevertheless, since their relative abundances are always higher than 0.8%, we define the fifteen genera above as those composing the rhizosphere core microbiome of our system. The theses in which the fungus is present show the specific presence of eight genera (*g_undetermined_Comamonadaceae*, *g_undetermined_Chitinophagaceae*, *Rubellimicrobium*, *Microvirga*, *Azohydromonas*, *Thauera*, *Microlunatus* and *Nakamurella*) accounting for a bacterial abundance in the 12–13% range. Compared to the control, it also appears that the introduction of *A. album* had a slight positive effect on the number of detectable genera (26 and 28 in the MX95 and N+MX95 theses, respectively, compared to 24 in the control). On the contrary, the presence of nematodes had a negative effect on the number of genera, which reached the lowest value of 18 in the N treatment. This led to the hypothesis that the presence of nematodes not only has negative effects on some plant traits but also is negatively correlated with the bacterial diversity of the associated rhizosphere.

**Figure 5:**
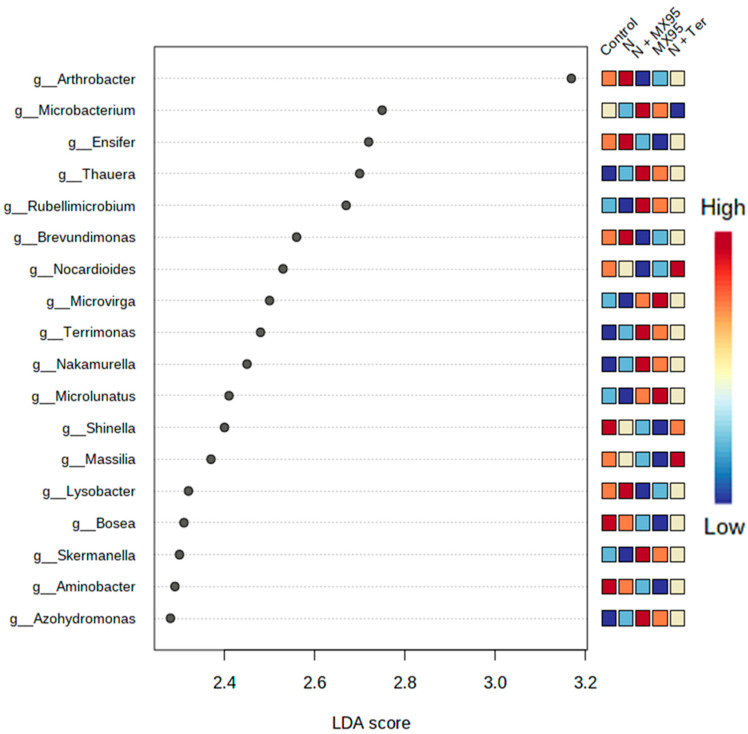
Lefse (LDA Effect Size) analysis. The dots show the genera with statistical differences among the treatments. The colours represent the relative abundances of the specific genera in each single treatment.

Finally, to estimate the size effect of each differentially abundant feature, an LDA effect size (LEfSe) analysis was performed (Figure 5) showing the specific genera that had significant differences among the treatments.

### 3.3. Pairwise Comparisons of Microbial Communities

In order to identify possible effects exerted on the rhizosphere microbiota by the different experimental conditions, the taxonomic profiles obtained for the five samples were analysed by pairwise comparisons. Table 4 reports the analysed pairwise comparisons together with the differentiation condition. The analysis was carried out by taking into account percentage variations of the genera abundances reported in Table S2.

In the pairwise comparison I, the presence of nematodes led to a lowering of the number of genera (from 24 to 18) together with relevant changes for some of the most abundant genera. For the genera *Paracoccus*, *Pseudomonas*, *Ensifer*, *Pseudoxanthomonas* and *AKIW781*, it was possible to observe large increases in relative abundances (33, 87, 28, 37 and 64%, respectively). Relevant reductions were detectable for the genera *Saccharimonadales*, *Nocardioides*, *g_undetermined_Xanthomonadaceae* and *Actinoplanes* by 29, 24, 29 and 52%, respectively. The genera *Shingobium* and *Streptomyces* of the core microbiome were no more detectable in the infested sample. Other genera not belonging to the core microbiome and no more detectable in the nematode infested treatment were *Shinella*, *g_uncultured_Saprospiraceae*, *Aminobacter*, *Massilia* and *g_undetermined_Oxalobacteraceae*. In the N treatment, only two specific genera (*Lysobacter* and *g_undetermined_Sphingobacteriaceae*) could be detected with relative abundances of 1.14 and 1.83%, respectively.

In the pairwise comparison II, the introduction of the *A. album* isolate had a slight positive effect on the number of detectable genera (from 24 to 26). Among the genera with an increase in relative abundances, *Paracoccus*, *AKIW781* and *Streptomyces* must be noted, with relatively large changes of 166, 131 and 136%, respectively. On the contrary, *Arthrobacter*, *Pseudomonas*, *Pseudoxanthomonas* and *Nocardioides* were the genera with the highest reductions (62, 59, 42 and 36%, respectively). A number of genera were specifically present in both the theses with *A. album* (*Rubellimicrobium*, *g_uncultured_Comamonadaceae*, *g_uncultured_Chitinophagaceae*, *Microvirga*, *Azohydromonas*, *Thauera*, *Microlunatus* and *Nakamurella*). Since their abundance in all the other theses was generally low (in the 0.00–0.50% range), an origin from the fungus cultures can be hypothesised.

**Figure 6:**
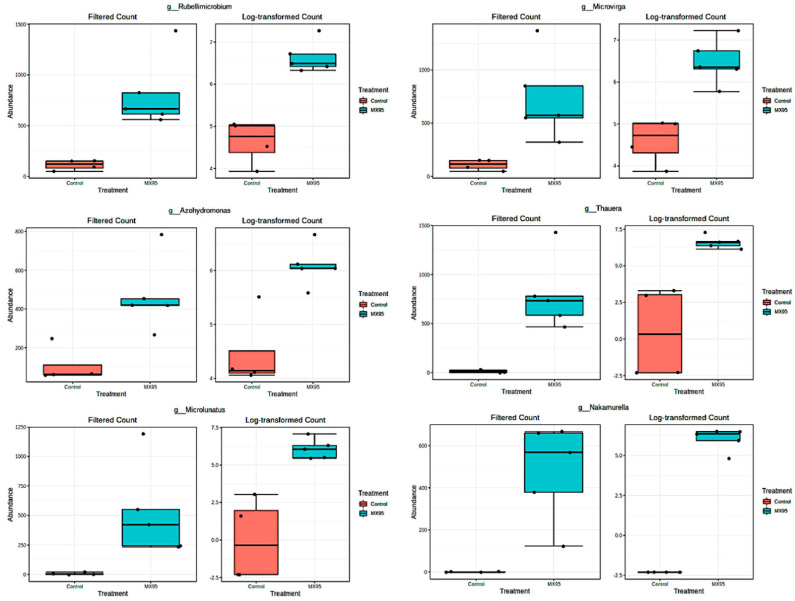
DESeq2 analysis referred to the pairwise comparison II.

For these specific genera, the comparisons of their abundances in the MX95 and control theses, as obtained by DESeq2 analysis, are reported in Figure 6.

The genera *Rubellimicrobium* [log2FC: 3.1933; *p* values: 9.45 × 10^−10^; FDR: 4.37 × 10^−8^], *Microvirga* [log2FC: 2.9824; *p* values: 4.78 × 10^−7^; FDR: 1.41 × 10^−5^], *Azohydromonas* [log2FC: 2.2898; *p* values: 0.00016101; FDR: 0.0026084], *Thauera* [log2FC: 6.6255; *p* values: 2.50 × 10^−11^; FDR: 2.70 × 10^−9^], *Microlunatus* [log2FC: 6.8272; *p* values: 1.13 × 10^−10^; FDR: 7.31 × 10^−9^] and *Nakamurella* [log2FC: 11.5791; *p* values: 1.15 × 10^−21^; FDR: 3.74 × 10^−19^] were considered.

In the pairwise comparison III, the treatment with *A. album* of nematodes-inoculated plants corresponded to large increases in abundance for five genera of the rhizosphere core microbiota (*Pseudoarthrobacter*, *Sphingomonas*, *Microbacterium*, *Saccharimonadales*, *Terrimonas* and *AKIW781*) with percentages in the 47–131% range. On the contrary, the other three genera of the core microbiota (*Arthrobacter*, *Pseudomonas*, *Ensifer* and *Pseudoxanthomonas*) decreased by 63, 77, 64 and 63%, respectively. The dynamics of the three genera *Pseudomonas*, *Pseudoxanthomonas* and *Ensifer* are interesting. These genera were found increased in the comparison of the control with the nematodes-inoculated sample (treatment 1 and 2) and all were found decreased in the nematocide treated sample (treatment 3). More interestingly, the same three genera decreased in the Tervigo treated sample (treatment 5 and the pairwise comparison IV below). Overall, it seems that these genera are positively correlated with the nematode presence. Additionally, the genera *Lysobacter* and *g_undetermined_Sphingobacteriaceae*, specifically associated with nematodes (see comparison I), were no more detectable in the nematicides treated theses (see also comparison IV). Inversely, the genus *Saccharimonadales*, which was found to be decreased after the introduction of the nematode and increased in the nematocides-treated samples (see also comparison VI), can be assumed as negatively correlated with nematodes.

In the pairwise comparison IV, the treatment of inoculated plants with Tervigo induced some large changes of genera abundances. Genera showing the largest variations were: Sphingomonas *(*+64%), *Paracoccus* (+64%), *Nocardioides* (+39%), *AKIW781* (+42%), *Actinoplanes* (+72%), *Pseudomonas* (−56%), *Ensifer* (−50%) and *Pseudoxanthomonas* (−47%).

## 4. Conclusions

In this work, we investigated the effects of the bionematicide fungus *A. album* MX-95 on plant morphological traits, nematode populations and the rhizosphere microbiome, in the case of tomato plants infested with the root-knot nematode *M. javanica*. While positive effects on some plant traits were not supported by statistical analysis, the fungus was effective in reducing the nematode total population and reproduction rate in the same way as the commercial nematicide Tervigo. An increased fungal population was also observed at the end of the experiments.

As for the effects on the composition of rhizosphere microbiome, the treatment with the bionematicide increased the number of detectable bacterial genera, especially in comparison with the rhizosphere of the *M. javanica* infested plants. The possible further effects of these changes on plant fitness constitute a topic of large interest for future investigations.

In conclusion, *A. album* can be favourably considered as a new bionematicide suitable to use in plant protection for control of *M. javanica*. However, further research is still needed to optimize product rates and methods of application after transplanting (e.g., dose and time) along with field validation experiments.

## Figures and Tables

**Table 1:** Plant growth parameters of *S. lycopersicum* cv. Regina plants cropped in *M. javanica* infested (N) or uninfested (Control) soil treated or untreated with *A. album* (MX-95) or the commercial nematicide Tervigo (Ter) ^1,2^.

Treatment	Shoot		Root
	Height (cm)	Dry Weight (g)	Weight (g)
Control	55.4 ± 10.2 ab	18.8 ± 2.5 a	80.2 ± 27.0 a
N	47.6 ± 7.5 a	16.9 ± 2.6 a	100.7 ± 31.5 ab
N + MX95	55.2 ± 3.3 ab	18.4 ± 2.7 a	166.6 ± 25.1 d
MX95	58 ± 4.1 b	19.0 ± 3.8 a	139.0 ± 9.3 cd
N + Ter	51.2 ± 4.6 ab	16.2 ± 1.5 a	113.3 ± 12.0 bc

^1^ Each value is the average of 5 replications ± SD; ^2^ data flanked in each column by the same letter are not statistically different according to the Least Significant Difference Test (*p* = 0.05).

**Table 2:** Nematological parameters of *S. lycopersicum* cv. Regina plants cropped in *M. javanica* infested (N) or uninfested (Control) soil treated or untreated with *A. album* (MX-95) or Tervigo (Ter) nematicide ^1,2^.

Treatment	RGI ^3^ (0–5)	Eggs + J2/Root System (N° × 1000)	Eggs + J2/g Root (N° × 100)	Eggs + J2/mL Soil (*Pf*)	Total Pop./mL Soil (Root + Soil) (Eggs + J2/mL Soil)	R = *Pf*/*Pi*
N	5.0 ± 0.0 a	203.6 ± 58.3 a	21.5 ± 7.1 b	6.1 ± 2.3 a	210 ± 56.9 a	280 ± 75.9 a
N+MX95	4.0 ± 1.0 ab	115.6 ± 36.6 b	6.9 ± 1.9 a	4.1 ± 2.6 a	120 ± 37.2 b	160 ± 50.3 b
N+Ter	3.2 ± 0.8 b	175.3 ± 29.4 b	15.7 ± 3.6 b	3.3 ± 1.1 a	179 ± 28.9 b	238 ± 38.3 b

^1^ Each value is the average of 5 replications ± SD; ^2^ data flanked in each column by the same letter are not statistically different according to the Least Significant Difference Test (*p* = 0.05); ^3^ RGI = Root Gall Index.

**Table 3:** Plate count of fungi from soil used in the pot trial of *S. lycopersicum* cv. Regina plants cropped in *M. javanica* infested (N) or uninfested (Control) soil treated or untreated with *A. album* MX-95.

Treatment	Colonies (N/g of Air-Dried Soil) ^1,2^	
	*A. album MX-95*	Other Fungal Species
Control	0 a	1.8 × 10^2^ ± 10 a
N	0 a	22 × 10^5^ ± 10 c
N + MX-95	44 × 10^9^ ± 2110 c	2 × 10^1^ ± 2 a
MX95	12 × 10^9^ ± 200 b	2 × 10^2^ ± 2 a
N + Ter	0 a	1.6 × 10^3^ ± 20 b

^1^ Each value is the means of 8 isolation plates ± SD; ^2^ in each column, data with the same letter are not statistically different according to the Least Significant Difference Test (*p* = 0.05).

**Table 4:** Pairwise comparisons and related different experimental conditions.

Pairwise	Investigated Treatment	Treatment Variation
I	N vs. Control	Nematodes
II	MX95 vs. Control	*A. album*
III	N + MX95 vs. N	*A. album*
IV	N + Ter vs. N	Tervigo

## Supplementary Materials

The following are available online at https://www.mdpi.com/2076-2607/8/12/1922/s1, Figure S1: Rarefaction curves of 16S rRNA ASVs for the treatment replicates; Figure S2: Summary of pairwise Dunn tests; Table S1: Denoising results for the different theses; Table S2: Relative abundances of bacterial genera obtained by metabarcoding analysis.
