# Supplementary material for: Plant Health and Rhizosphere Microbiome: Effects of the Bionematicide Aphanocladium album in Tomato Plants Infested by Meloidogyne javanica
Source: Microorganisms. 2020 Dec 3;8(12):1922. doi: 10.3390/microorganisms8121922 (PMC7761678; doi:10.3390/microorganisms8121922)
Supplement: Supplementary file 1 [file microorganisms-08-01922-s001.pdf]

SUPPLEMENTARY MATERIALS

Plant health and rhizosphere microbiome: effects of the bio-nematicide *Aphanocladium album* in tomato plants infested by *Meloidogyne javanica*

Claudia Leoni[1,a], Elisabetta Piancone[2,a], Nicola Sasanelli[3], Giovanni Luigi Bruno[4], Caterina Manzari[1], Graziano Pesole[1,2,5], Luigi R. Ceci[1,*] and Mariateresa Volpicella[1,2,5,*]

1 Institute of Biomembranes, Bioenergetics and Molecular Biotechnologies, CNR, Via Amendola 165/A, 70126 Bari, Italy; c.leoni@ibiom.cnr.it C.L.; e.piancone@ibiom.cnr.it (E.P.); c.manzari@ibiom.cnr.it (C.M.)
2 Department of Biosciences, Biotechnologies and Biopharmaceutics, University of Bari Aldo Moro, Via Amendola 165/A, 70126 Bari, Italy; graziano.pesole@uniba.it (G.P.)
3 Institute for Sustainable Plant Protection, CNR, Via G. Amendola 122/D, 70126 Bari, Italy; nicola.sasanelli@ipsp.cnr.it (N.S.)
4 Department of Soil, Plant and Food Sciences, University of Bari Aldo Moro, Via Amendola 165/A, 70126 Bari, Italy; giovanniluigi.bruno@uniba.it (G.L.B.)
5 Consorzio Interuniversitario Biotecnologie, Località Padriciano, 99, 34149 Area di Ricerca, Trieste, Italy.

* Correspondence: mariateresa.volpicella@uniba.it (M.V.); l.ceci@ibiom.cnr.it (L.R.C.)
[a] These authors have contributed equally to this work.

List of material:

Table S1.: Denoising results for the different treatments.
Table S2.: Relative abundances of bacterial genera obtained by metabarcoding analysis.

Figure S1: Rarefaction curves of 16S rRNA ASVs for the treatment replicate**s.**
Figure S2.: Summary of pairwise Dunn tests.

Table S1: Denoising results for the different treatments.
Biological replicates are indicated in the sample-id column as 1 for Control thesis, as 2 for N thesis, as 3 for N+MX95, as 4 for MX95 and as 5 for N+Ter.

Sample-id	Input	Filtered	Percentage of input passed filter	Denoised	Merged	Percentage of input merged	Non-chimeric	Percentage of input non-chimeric
1-1A	46040	39979	86.84	39222	36064	78.33	35354	76.79
1-1B	57801	51910	89.81	51187	48797	84.42	47701	82.53
1-1C	60760	54078	89	52945	49153	80.9	48417	79.69
1-2A	62936	56031	89.03	54982	51317	81.54	50382	80.05
1-2B	58446	51521	88.15	50678	47343	81	44795	76.64
2-1A	78218	67336	86.09	66352	62249	79.58	61241	78.3
2-1B	67927	60301	88.77	59431	56404	83.04	55737	82.05
2-1C	65274	57338	87.84	56365	53089	81.33	51569	79
2-2A	76139	66499	87.34	65482	61625	80.94	61075	80.22
2-2B	81391	70918	87.13	69982	66104	81.22	64392	79.11
3-1A	77833	68532	88.05	67589	63797	81.97	62448	80.23
3-1B	59237	52270	88.24	51189	46858	79.1	45988	77.63
3-1C	59483	50255	84.49	49207	44416	74.67	43705	73.47
3-2A	76806	67893	88.4	66875	62078	80.82	60566	78.86
3-2B	62206	55460	89.16	54390	50153	80.62	49224	79.13
4-1A	57175	50491	88.31	49469	45393	79.39	44626	78.05
4-1B	74269	66644	89.73	65535	61042	82.19	60323	81.22
4-1C	82941	74029	89.26	73009	68610	82.72	67174	80.99
4-2A	77642	69605	89.65	68431	64134	82.6	63147	81.33
4-2B	72595	64742	89.18	63800	59129	81.45	56860	78.32
5-1A	72939	63600	87.2	62336	57731	79.15	56696	77.73
5-1B	72085	63835	88.56	62950	59479	82.51	58165	80.69
5-1C	73715	64042	86.88	62892	57907	78.56	56367	76.47
5-2A	78497	69366	88.37	68612	64763	82.5	62823	80.03
5-2B	77856	68205	87.6	67055	63313	81.32	62564	80.36
Mean	69208.44	60995.2	89	59998.6	56037.92	81	54853.56	79
DS	9494,36	8432,86	1,23	8398,33	8196,70	1,95	8080,38	2,01

Table S2. Relative abundances of bacterial genera obtained by metabarcoding analysis.
ASVs which could not be resolved at the genus level were reported with the notation g_undetermined followed by the name of the closest known parental rank.

**Genus**	**Thesis**				
	**Control**	**N**	**N + Ter**	**N + MX95**	**MX95**
Arthrobacter	11.02	11.23	10.26	4.20	4.15
Pseudarthrobacter	5.38	5.15	5.67	8.05	6.16
Nocardioides	3.83	2.89	4.02	2.40	2.45
Pseudomonas	3.35	6.28	2.79	1.47	1.38
Microbacterium	2.83	2.61	2.16	5.02	3.50
Ensifer	2.75	3.50	1.75	1.26	0.95
Pseudoxanthomonas	2.70	3.70	1.95	1.39	1.57
Streptomyces	2.27	0.84	1.48	2.94	5.35
Actinoplanes	2.26	1.09	1.88	0.90	0.53
Paracoccus	2.22	2.95	4.84	2.26	5.90
Sphingomonas	2.13	1.93	3.17	2.85	2.80
Saccharimonadales	2.09	1.48	1.75	2.34	2.79
g_undetermined_Xanthomonadaceae	1.80	1.27	0.77	0.49	0.64
Shinella	1.78	0.85	0.91	0.71	0.70
Brevundimonas	1.73	1.84	1.46	0.06	0.23
Bosea	1.54	1.42	1.10	0.75	0.57
g_undetermined Micrococcaceae	1.49	1.38	1.18	1.67	1.78
Sphingobium	1.35	0.80	1.15	1.47	1.28
g_uncultured_Saprospiraceae	1.25	0.77	0.93	1.10	1.75
Aminobacter	1.23	0.58	0.49	0.42	0.28
AKIW781	1.19	1.95	2.78	3.02	2.76
Massilia	1.09	0.97	1.48	0.51	0.35
Bdellovibrio	1.08	1.16	0.97	0.63	0.82
g_undetermined Oxalobacteraceae	1.04	0.57	0.70	0.32	0.26
Terrimonas	0.98	1.01	1.03	2.34	1.83
g_uncultured_Blastocatellaceae	0.91	0.94	1.19	0.89	1.01
Devosia	0.87	0.75	1.01	0.74	0.54
g_undetermined Sphingomonadaceae	0.85	0.66	1.10	1.27	1.31
Cupriavidus	0.80	0.99	0.52	1.12	0.37
Lysobacter	0.76	1.14	0.65	0.13	0.28
g_undetermined Comamonadaceae	0.50	0.86	0.81	1.47	1.05
g_undetermined Chitinophagaceae	0.43	0.64	0.78	1.62	1.82
g_undetermined Sphingobacteriaceae	0.39	1.83	0.83	0.14	0.17
Rubellimicrobium	0.29	0.13	0.61	2.36	1.93
Microvirga	0.26	0.18	0.31	1.77	1.73
Azohydromonas	0.24	0.43	0.76	1.18	1.12
LWQ8	0.22	0.87	1.20	1.29	1.11
Skermanella	0.09	0.06	0.08	1.00	0.98
Thauera	0.03	0.08	0.19	2.46	1.82
Microlunatus	0.02	0.01	0.16	1.10	1.23
Nakamurella	0.00	0.01	0.01	1.43	1.17
Others	32.99	32.19	33.13	31.47	31.60

Figure S1: Rarefaction curves of 16S rRNA ASVs for the treatment replicates.

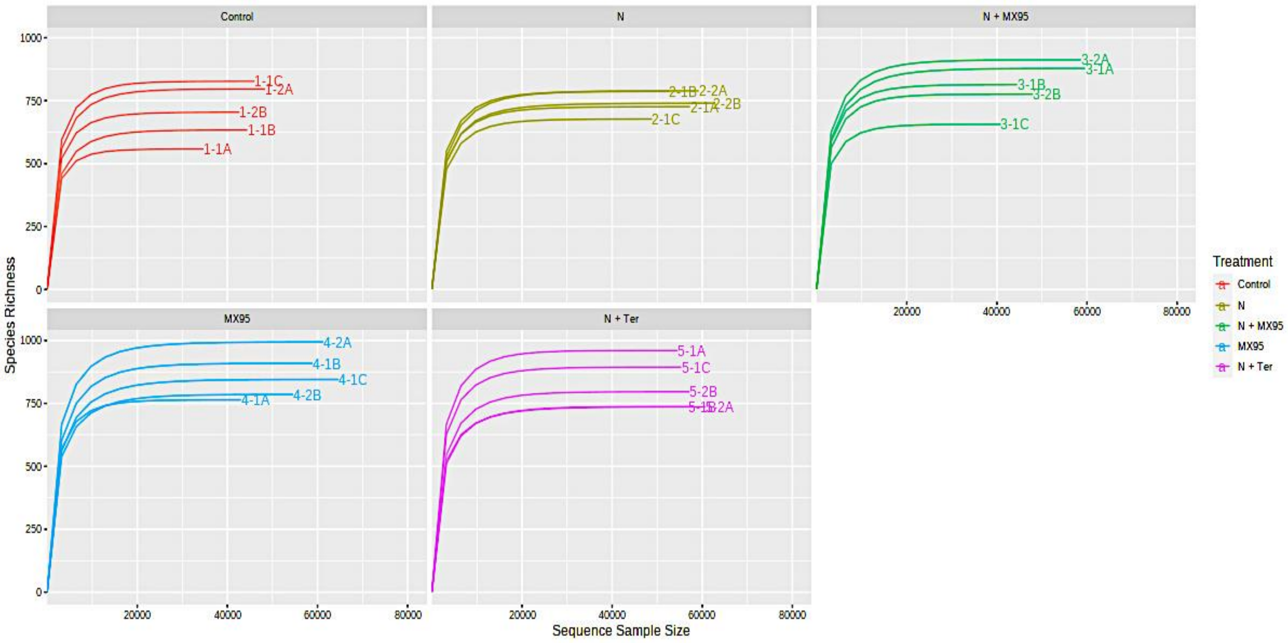

The name reported in the graphics for each curve indicates the biological replicate for each thesis.

Figure S2: Summary of pairwise Dunn tests.

```
Kruskal-Wallis chi-squared = 9.881, df = 4, p-value = 0.04

                           Comparison of x by group
                                 (Bonferroni)
Col Mean-|
Row Mean |    Control       MX95          N   N + MX95
---------+--------------------------------------------
    MX95 |  -1.022469
         |     1.0000
         |
       N |   1.296533   2.459674
         |     0.9740     0.0695
         |
N + MX95 |  -1.359779  -0.357770  -2.817445
         |     0.8695     1.0000    0.0242*
         |
 N + Ter |  -0.685160   0.357770  -2.101903   0.715541
         |     1.0000     1.0000     0.1778     1.0000

alpha = 0.05
Reject Ho if p <= alpha/2
```
